# Communication of Uncertainty about Preliminary Evidence and the Spread of Its Inferred Misinformation during the COVID-19 Pandemic—A Weibo Case Study

**DOI:** 10.3390/ijerph182211933

**Published:** 2021-11-13

**Authors:** Jiahui Lu, Meishan Zhang, Yan Zheng, Qiyu Li

**Affiliations:** 1 State Key Laboratory of Communication Content Cognition, People’s Daily Online, Beijing 100733, China; 2 School of New Media and Communication, Tianjin University, Tianjin 300072, China; mason.zms@gmail.com (M.Z.); yanzheng@tju.edu.cn (Y.Z.); tjliqy@tju.edu.cn (Q.L.); 3 Wee Kim Wee School of Communication and Information, Nanyang Technological University, Singapore 637718, Singapore; 4 School of Computer Science and Engineering, Nanyang Technological University, Singapore 639798, Singapore

**Keywords:** social media, COVID-19, misinformation, uncertainty, preliminary evidence

## Abstract

The rapid spread of preliminary scientific evidence is raising concerns on its role in producing misinformation during the COVID-19 pandemic. This research investigated how the communication of uncertainty about preliminary evidence affects the spread of its inferred misinformation in a Weibo case study. In total, 3439 Weibo posts and 10,380 reposts regarding the misinformation of pets transmitting COVID-19 were analyzed. The results showed that attitude ambiguity toward the preliminary evidence and the stage when the evidence was first released with uncertainty were associated with higher numbers of likes and retweets of misinformation posts. Our study highlights the internal sources of misinformation and revisits the contextual perspective in misinformation studies.

## 1. Introduction

The coronavirus disease pandemic (COVID-19) has triggered a “misinfo-demic” that warrants continuous research efforts [1]. Misinformation involving various topics has emerged and posed harm to people’s lives [2,3]. Notably, social media has facilitated the spread of misinformation in this unprecedented pandemic [4,5]. Over a quarter of social media information has been found to contain medical falsehoods and unverified, low-quality content about COVID-19 [6]. Many mechanisms afforded by social media platforms have been argued to support such a misinformation pandemic [7,8].

In this study, we aim to offer a new theoretical framework to explain the misinfo-demic by focusing on internal sources of misinformation and factors that facilitate the misinformation spread on social media. First, we theorize the rapid dissemination of preliminary scientific evidence on social media as a context where scientific misinformation can arise due to individuals’ naïve understanding of science. For example, the release of evidence that a pet dog tested “weak positive” has fueled the widespread misinformation that pets can transmit COVID-19 [9]. Though this issue is not new, the COVID-19 pandemic has amplified the adverse consequences of such a hasty science communication process on social media. Research has expressed a worry about the surge in the use of preliminary COVID-19-related evidence by media outlets and its role in driving the ongoing COVID-19 discourse [10].

Second, we aim to examine how communicating uncertainty about preliminary evidence affects the spread of COVID-19 misinformation and its refutations. Given the fast-developing nature of science during the pandemic, the communication of preliminary evidence is often swiftly evolving, from communicating with no evidence, evidence that is first released with uncertainty on its interpretations, to evidence that has reached some consensus from the science community. In addition, scientists often approach preliminary evidence with caution, which may signal uncertainty of the evidence. Therefore, it is essential to investigate whether and how uncertainty communicated surrounding preliminary evidence will be a factor that facilitates or inhibits the spread of misinformation.

### 1.1. Misinformation Generation and Spreading

Research has found different sources of misinformation. Lewandowsky et al. extensively discussed four sources of misinformation, including rumors and fiction, governments and politicians, vested interests, and the media [11]. In the COVID-19 pandemic, low-quality preprints are also found to be an important driver of the misinfo-demic [10]. While studies focused largely on the above external resources from the environment that disseminate false information, Coronel, Poulsen, and Sweitzer reveal that memory biases and distortions of accurate information are an internal source of misinformation [12], in which misinformation is generated through individuals’ memory processes after their exposure to factually accurate information [13].

Studies also reveal various content, sender, and network factors that can fuel misinformation spread. For example, content topic and novelty facilitate the spread of false news online [14]. Senders’ feelings of uncertainty, anxiety, trust in the information source, perceived importance of the information, cultural beliefs, and motivations are all associated with misinformation spreading, corresponding to and extending Allport and Postman’s “basic law” of rumor transmission [15,16] (see a review in [11]). Research also finds that echo chambers play an important role in misinformation diffusion networks [17,18]. 

While contributions of existing scholarship are essential, they suffer from two significant gaps. First, research on internal sources of misinformation is scarce. Though Coronel et al. [12] reveal memory biases as an internal source of misinformation, they assume a context where people are exposed to factually accurate information from the environment. However, in health crises such as the COVID-19 pandemic, much information is preliminary and uncertain when it is first communicated with the public, and it is observed that misinformation can naturally arise from the preliminary information. For example, when Hong Kong released the scientific evidence that a dog tested “weak positive” for the virus without confirmation of its infection, the misinformation that pets could transmit COVID-19 to humans rapidly spread and triggered irrational actions such as abandoning or killing pets as a precaution [9]. Within the example, the information from the external source is from an authority and is factually accurate but preliminary, and the misinformation is unlikely derived from individuals’ memory biases. The current scholarship of misinformation has yet to offer what internal processes can cause such a phenomenon to occur and thereby the corresponding solutions.

Second and relatedly, as most studies assume sources of misinformation as external, they focus on attributes of misinformation and individual characteristics that facilitate the misinformation spread. As such, there is a lack of answers to questions about what are the contextual prerequisites for individuals to produce and spread misinformation. Though Rosnow pointed out as early as in 1988 that it is vital “to decipher the experiential contexts that ‘invite’ or ‘allow’ rumors to flourish [19] (p. 16),” this proposal has not attracted much research attention thus far in the misinformation literature. However, the proposal is fundamental, as it will direct a new research approach upon strategies for building an appropriate communication environment that reduce internal sources of misinformation. Such an approach can prevent misinformation from being generated and spread, and thereby complement the current correction-based approach on misinformation after the misinformation has been spreading. This prevention-based approach is significant for health policymakers and crisis communicators as it puts communication efforts before the misinformation emerges and spreads and can reduce the potential detrimental impacts of misinformation on the society. 

To fill the above gaps, this study focuses on another internal source of misinformation: the instance in which individuals are exposed to preliminary scientific evidence from authorities, but naïve understanding of science causes them to misinterpret the preliminary information. We focus on the spread of this type of misinformation especially in the COVID-19 pandemic, where individuals lack prior knowledge on the disease and are motivated to reduce uncertainty. In the following sections, we aim to outline the theoretical foundation of how preliminary evidence can become a prompt for individuals to generate misinformation from internal processes and what information context can facilitate such processes of misinformation generation and spreading. 

### 1.2. Misinterpreting Preliminary Evidence Based on Naïve Theories of Science

We theorize the phenomenon of misinformation arising from preliminary scientific evidence from a socio-cognition perspective on the public understanding of science. According to the concept of epistemic cognition, intuitive and naïve theories of science provide a basic orientation toward scientific information [20]. Specifically, the general public can often process only one-sided evidence and lack an understanding of how much evidence is needed to justify a scientific claim [21,22]. As such, when a piece of preliminary evidence emerges, the public may infer scientific claims that can be unjustified by the evidence. This type of inferred misinformation might be particularly prominent during a novel health crisis such as the COVID-19 pandemic, as people rely more on general epistemic beliefs in science when their understanding of the subject is lacking [23,24]. Though the inferred information may not always be false, communication about early science and preliminary research can potentially form a context where scientific misinformation can arise and thus needs imperative research attention. 

In addition, refuting the inferred misinformation is often challenging. Debunking messages of the evidence-inferred misinformation will often require detailed explanations of mechanisms that are still unclear and under investigation. Additionally, as the misinformation is often an unjustified scientific claim, it cannot be truly refuted as false. Instead, science can only argue that there is no support for such a claim on most occasions. Furthermore, the refutation will easily elicit the audiences’ negative feelings, as it will unavoidably interrupt a logical and coherent inference based on lay beliefs in science [25]. 

The above presents a highlighted paradox of science communication in the pandemic. When the preliminary science is communicated, audiences will potentially infer logically sound (at least from epistemic beliefs of the general public) but unjustified scientific misinformation for guiding future actions. As such, an initial investigation on whether and how communication of preliminary evidence can motivate people to generate and spread the inferred misinformation offers valuable insight into strategies that can tackle this type of misinformation. 

### 1.3. Evidence Uncertainty as an Information Context for Misinformation Spread

As mentioned, few studies have examined what information context can facilitate the generation and spreading of misinformation. We propose that evidence uncertainty is one of the possible facilitators. Uncertainty “exists when details of situations are ambiguous, complex, unpredictable, or probabilistic; when information is unavailable or inconsistent; and when people feel insecure about their own state of knowledge or the stage of knowledge in general [26] (p. 478).” Crisis communication research has emphasized the importance of uncertainty reduction and timely communication [27,28]. However, balancing certainty with urgency is challenging. This is particularly the case in the COVID-19 pandemic as it is common, and often expected, to communicate timely, preliminary evidence that has not gained scientific consensus and certainty [29,30]. 

Here, at least two types of uncertainty will be involved in the communication of preliminary findings. First, preliminary evidence often lacks sufficient data and has limitations in addressing a novel crisis phenomenon and thus signals deficient uncertainty about a known gap [31,32]. Second, agreement in interpreting the evidence may not be reached by scientists and other stakeholders as it was first released, which creates consensus uncertainty. Past literature has found that the communication of deficient uncertainty and consensus uncertainty can affect people’s trust in science and intention to follow recommendations [29,33]. However, no studies have investigated how the communication of evidence uncertainty can be related to the spread of misinformation. 

According to the concept of motivated reasoning [34,35], ambiguous risk messages will motivate people to access and construct information in a heuristic way to reduce uncertainty. Research has supported this conceptual notion by showing that the communication of evidence uncertainty accentuates the reliance on people’s own experiences, heuristics, and feelings about risk, and the disregard of institutional assessments [36,37]. For example, conflicting interpretations of research evidence regarding cancer risks were found to trigger people’s dispositional beliefs in cancer fatalism [38]. In addition, a recent systematic review of 48 experimental studies revealed that the communication of uncertainty regarding the evidence’s deficiency and consensus yielded adverse effects in terms of decreasing belief in, perceived credibility of, or intentions to follow recommendations of the message in most risk communication research [39]. Importantly, the crisis environment may further provoke people’s inclination to “seize” and “freeze” the certainty developed through their heuristic cognition [25]. Anxiety and aversion induced by the environment can heighten people’s desire to form and maintain a quick and clear-cut judgment that can protect them from the crisis even when such a judgment may be false [40,41].

### 1.4. Research Framework

Based on the above review, we propose a new framework that the uncertainty communicated with preliminary evidence can promote internal motivated reasoning based on a naïve understanding of science that produces misinformation inferred from the evidence. That is, given that people rely more on heuristic reasoning when facing uncertainty in a crisis, communication about the preliminary evidence with uncertainty should motivate people to interpret the evidence based on their naïve beliefs, thus likely resulting in the inferred misinformation. As discussed, refutations of the inferred misinformation often contain detailed explanations that require high-level processing efforts. Additionally, refuting messages may even introduce more uncertainty as mechanisms underlying the evidence may still be unknown. In comparison, inferring (mis)information from the evidence based on epistemic beliefs requires fewer cognitive efforts, which may be a better way for people to achieve some extent of certainty in a novel crisis. Therefore, people may favor misinformation and be averse to its refutations in the context where the uncertainty of preliminary evidence is communicated. 

### 1.5. Research Hypotheses

To test our theoretical framework, we examine if the communication of preliminary evidence could be a prompt for individuals’ internal processes to generate misinformation and if communicating the uncertainty of the evidence would facilitate misinformation spread based on social media data. First of all, if the communication of preliminary evidence indeed prompts individuals’ processes to generate misinformation, social media users’ attitudes toward the evidence should be associated with their attitudes toward the inferred misinformation. Therefore, we hypothesize that:

**Hypothesis** **1** **(H1).** *Users’ attitudes toward preliminary evidence will be associated with their attitudes toward misinformation*.

In addition, we examine the communication of evidence uncertainty in two manifestations. First, we test if social media users’ attitude ambiguity towards the evidence would be associated with the inferred misinformation spread. Scientists and health professionals often caution with the preliminary evidence and thereby demonstrate attitude ambiguity when it is first released. This is especially the case during the COVID-19 pandemic. However, it is unknown how such attitude ambiguity toward the evidence may affect users’ responses to the inferred misinformation and the refutations. Based on the literature reviewed above, we hypothesize that:

**Hypothesis** **2** **(H2).** *Attitude ambiguity on preliminary evidence will be associated with users’ preferences for misinformation messages and aversion to refutations*.

Second, we compare social media users’ responses to misinformation and refutation messages across different evidence communication stages. Notably, preliminary evidence is often released with cautions about its interpretations by authorities or scientific professionals. Thus, uncertainty is inherent in the preliminary evidence when it is first communicated. It is only based on further investigations or recognitions from the science community that the preliminary evidence can gain some scientific consensus. In this case, early science communication will naturally unfold three communication stages, from no evidence, uncertain evidence, to evidence consensus. We hypothesize that:

**Hypothesis** **3** **(H3).** *The uncertain-evidence stage will be associated with users’ preferences for misinformation messages and aversion to refutations.*

Third, we explore how the two manifestations of evidence uncertainty communication interact to affect users’ responses to misinformation and refutation messages. We do not assume the two forms of evidence uncertainty would have the same effect as they may indicate different levels and types of uncertainty. Thus, we ask a research question (RQ):

**RQ:**  *How do the two forms of evidence uncertainty communication interact to affect people’s responses to misinformation and refutation messages on social media*?

We tested the above hypotheses using social media data on Weibo. Social media offers a valuable data source that can naturally unfold the generation and spread of misinformation. For our investigation, we focus on the scientific misinformation about pets transmitting COVID-19 to people. No evidence has been found today to support the misinformation. However, evidence that pets could be infected with the virus was accumulating during the early stage of the pandemic and has fueled misinformation and irrational public panic. Particularly, Hong Kong reported the first instance that a dog of a COVID-19 patient tested “weakly positive” for the virus on 28 February 2020. Notably, when this news was first released, the Hong Kong scientists emphasized that they were still unsure if the dog was actually infected or just contaminated by the environment. It was only on 4 March that scientists from the WHO concluded it is a case of human-to-animal transmission of the virus. This context manifests the communication process of early science from communication with no evidence (i.e., before 28 February), uncertain evidence (i.e., 28 February to 3 March), and evidence consensus (i.e., 4 March onward). Thus, it offers an appropriate research setting for our examination.

Particularly, we examine users’ responses regarding their liking and reposting of misinformation posts. Numbers of liking and reposting have been found to be associated with rumor-spreading behaviors on social media platforms such as Twitter. Alhabash and McAlister defined the number of retweets as a manifestation of the viral reach of a message, and the number of likes as a manifestation of the affective evaluation of the message [42]. Both indexes can serve as normative cues that increase a given rumor’s perceived creditability and induce users’ intention to share the rumor [43]. In addition, we also examine users’ attitudes toward misinformation in a repost as an indicator of misinformation responses. Past misinformation literature focused predominantly on numbers of reposts regardless of their authors’ attitudes toward the misinformation [43]. However, a high number of reposts debunking the misinformation can help misinformation rebuttal instead.

## 2. Materials and Methods

### 2.1. Data Collection

Weibo posts in the Chinese language (traditional and simplified Chinese) related to the misinformation of pets transmitting COVID-19 to humans were collected using Weibo’s application programming interface (API). Data collection was conducted on 19 May 2020 using a retroactive keyword sourcing dating back to 1 January 2020, when the infections were first reported to the WHO. Search keywords included three sets of Chinese-language terms: (1) pet-related keywords: [宠物] (pet), [猫] (cat), [狗] (dog), [家养动物] (domestic animal); (2) transmission-related keywords: [传染] (infection, infect, transmit, transmission), [传播] (spread), [宿主] (host); and (3) COVID-19-related keywords: [冠状病毒] (coronavirus), [新冠, 新冠病毒, 新型冠状病毒] (novel coronavirus), [肺炎] (pneumonia), [新型肺炎] (novel pneumonia), [新冠肺炎] (novel coronavirus pneumonia), [武汉肺炎] (Wuhan pneumonia), nCov, and COVID-19. These keywords were chosen based on the public conversations related to the misinformation. A total of 3439 original Weibo posts that contain keywords from each of the three keyword sets and 10,380 reposts from those original posts were collected. Note that the data were only a collection of the full data pool based on the Weibo API rule. 

### 2.2. Measurements

#### 2.2.1. Users’ Attitudes toward the Evidence and the Misinformation

**Figure 1:**
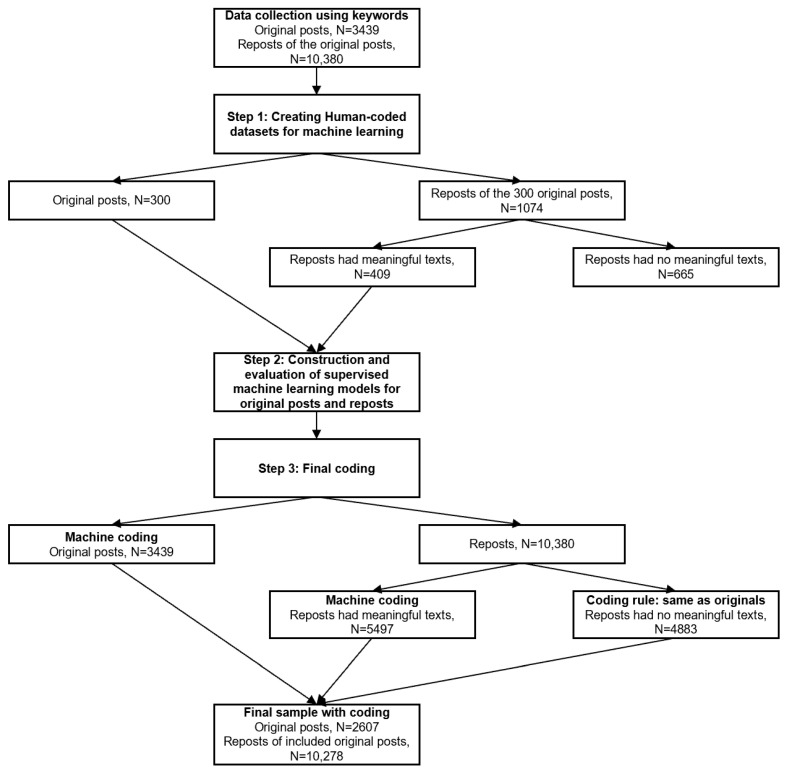
The data flow.

We employed a machine learning approach to measure users’ attitudes toward the evidence and the misinformation in the Weibo posts and reposts. This process included three steps (see Figure 1 for the data flow).

Step 1: Creating human-coded datasets.

In the first step, two human-coded datasets were created for the original Weibo posts and the reposts. A randomized subsample of 300 unduplicated original Weibo posts and their 409 reposts with meaningful texts (e.g., original texts, texts from the original posts) was constructed for human annotation. Please see step 3 for the coding logic for reposts that did not have meaningful texts. 

Coding dataset for the original posts. Two statements were coded in the annotation process for the original post dataset: 

Misinformation statement: “Pets can transmit COVID-19 to people.” This includes statements that pets, or domestic animals, can transmit the infection to humans, or simply that pets can transmit COVID-19 without mentioning a proper object. 

Evidence statement: “Pets can be infected with the virus.” This includes statements that pets, or domestic animals, have been infected with the coronavirus or pets tested positive for the virus infection or other similar statements. 

Three coders, who were required to read a coding instruction and go through a training process before labeling, independently indicated the author’s attitudes toward the two statements for each post into the following four categories: (1) endorsing: posts repeating or confirming the misinformation; (2) rejecting: posts denying the misinformation or citing those who debunked the misinformation; (3) ambiguous: posts relevant to the statement but not showing perceivable attitudes toward it; (4) not mentioned: all other posts not mentioning or relevant to the statement. Krippendorff’s alpha tests revealed an acceptable level of intercoder reliability for the two statements: 0.75 for the misinformation statement and 0.71 for the evidence statement. The coding disagreement was resolved by discussions. Note that an author could have different attitudes towards the two statements in a single post. If original posts were not irrelevant to both statements, they were excluded from further analyses. 

Coding dataset for the reposts. Three coders annotated the author’s attitude toward the misinformation statement in a repost into the four categories mentioned above and meanwhile were presented with the text of its original post for reference. The Krippendorff’s alpha was 0.74, indicating an acceptable level of intercoder reliability. Disagreements were resolved by discussions.

Step 2: Construction and performance of supervised machine learning models. 

Label annotation was a classification problem. We used the pre-trained Chinese Bidirectional Encoder Representations from Transformers (BERT) models to build our text classifiers. BERT has been widely used in natural language processing as it obtained state-of-the-art performances in a wide range of tasks [44]. The model provides pre-train parameters of domain-general syntactic and semantic features that can be fine-tuned with labeled data for the domain-specific classification task.

To construct classifiers, we first pre-processed the data by removing text elements such as mentions (i.e., @username) and hyperlinks and segmenting raw texts into words with the Chinese lexical analyzer Jieba. Then, we randomly partitioned the human-coded original posts or reposts dataset into a training set (70%), a development set (20%), and a test set (10%). The training set was used to build the fine-tuned BERT models. The development set was used to evaluate the accuracy of variations of the models. Once the models achieved adequate performance on the development data, the test set was used to evaluate the final performance of the classifiers based on classification accuracy. We conducted the above processes for both original posts and reposts datasets. For the classifier regarding the original post dataset, the raw accuracy was 0.82 for the misinformation statement and 0.91 for the evidence statement. For the classifier regarding the repost dataset, the raw accuracy was 0.74. Therefore, the obtained machine-learning-based annotation accuracy was adequate for full dataset annotations with acceptable noise. 

Step 3: Final annotations. 

For original posts, we used the built classifiers to label the entire dataset. For reposts, we included only reposts of the relevant original posts into the final annotation and separated them into two subsets. The first subset contains reposts with meaningful texts such as original texts and texts from the original posts. This subset was annotated with the built classifiers. The second subset contains reposts that did not have meaningful texts, such as “repost” or “null” text. This subset was coded as the same attitude as that of the original posts toward the misinformation statement.

#### 2.2.2. Evidence Stage

The evidence stage was measured based on the timeline of evidence communication. As discussed, Hong Kong reported the first instance that a dog of a COVID-19 patient tested “weakly positive” for the virus on 28 February 2020; on 4 March, scientists from the WHO concluded it is a case of human-to-animal transmission of the virus. Thus, the evidence stage was identified as the three following stages: no evidence (i.e., before 28 February), uncertain evidence (i.e., 28 February to 3 March), and evidence consensus (i.e., 4 March onward).

### 2.3. Analysis Strategies

Our analyses included three dependent variables: numbers of likes and reposts, and numbers of reposts in different attitudes toward the misinformation. For the total number of likes and reposts, all relevant original posts were analyzed. For the number of reposts in different attitudes, only original posts that have reposts collected in the dataset were analyzed. Given that the dependent variables were frequency types of data and violated the normality assumption of standard parametric tests, we conducted a series of nonparametric tests for the analysis. 

For H1, we employed a chi square test to examine the association between attitudes toward the evidence and those toward the misinformation. For H2 and 3 and RQ, we used the Aligned Rank Transform (ART) technique for nonparametric factorial analysis in the tests. The ART technique is a ranked-based test procedure that first assigns a rank to each raw data point in an increasing order and then conducts regular parametric factorial tests on those ranks [45]. It is robust for small sample sizes (e.g., N = 2 per cell) and unbalanced sample sizes [46]. In addition, the ART program provides contrast tests comparing differences of pairwise combinations of levels between factors and those comparing differences of differences. It thus offers various ways to interpret the interaction effect between factors [47]. However, the ART program did not output the estimated mean ranks of each combination of levels. Therefore, we calculated the relative mean ranks based on the output of the differences of pairwise comparisons between cells. We always assigned the lowest averaged ranks for all analyses as 0 value as the baseline for comparison. All ART analyses were conducted in R software with the ARTool Package [45].

## 3. Results

### 3.1. Data Overview

**Figure 2:**
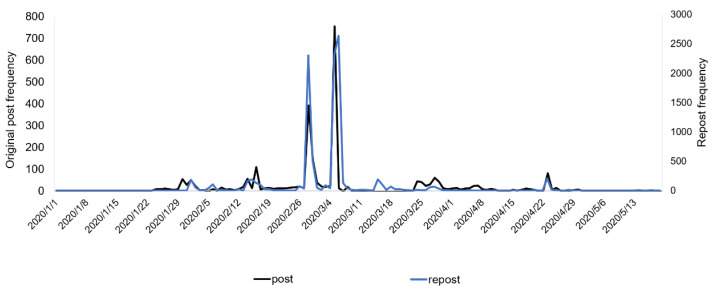
Numbers of posts and reposts regarding pets transmitting COVID-19 to humans across time.

A total of 2607 (75.8%) original posts were coded as relevant to the misinformation discourse and included in the analysis. Among them, 570 posts received a total of 10,278 reposts. These reposts were also included in the analysis. Figure 2 demonstrates the number of relevant posts and reposts across time. Both original posts and reposts witnessed the first peak on 28 February when Hong Kong released the preliminary evidence of a pet dog testing “weakly positive” for the virus, and the largest peak at 5/6 March, immediately after the infection was confirmed on the night of 4 March. This supports our measurement of the evidence stage. Note that the misinformation began to circulate in a small amount on Weibo before any evidence was presented. 

We observed a low endorsement rate (3.6%) and a high rejection rate (74.1%) of the misinformation for original posts. In addition, we found a high endorsement rate (78.0%) of the evidence. We observed a rate of 6.51% for reposts in endorsing the misinformation and 58.9% in rejecting it. Furthermore, approximately one-fourth (25.1%) of the reposts were coded as irrelevant to the misinformation. 

Table 1 presents the crosstab frequency table of authors’ attitudes toward the misinformation and its evidence in the original posts. Supporting H1, attitudes toward the misinformation and the evidence were correlated, *χ*^2^(9) = 257.6, *p* < 0.001. Notably, users of 65.9% (91 out of 138) of posts rejecting the evidence rejected the misinformation, and those of 72.0% (36 out of 50) of posts being ambiguous about the evidence were also uncertain about the misinformation. For users who endorsed the evidence in posts, 76.3% (1552 out of 2034) also rejected the misinformation, and only 2.3% (46 out of 2034) supported the misinformation. Within users who endorsed the misinformation in posts, 49.5% (46 out of 93) also endorsed the evidence. This suggests that users’ attitudes toward the misinformation and the evidence generally corresponded. Nevertheless, users of posts who rejected the misinformation were also likely to endorse the evidence in our dataset. 

We organized the data into three evidence stages: no evidence (1 January to 27 February), uncertain evidence (28 February to 3 March), evidence consensus (4 March on-ward) (see Table 1). At the no-evidence stage, the misinformation was circulated mainly without reference to the evidence (61.2%, 150 out of 245). If the evidence was mentioned, users likely rejected the evidence (24.1%, 59 out of 245). Their belief in the evidence and the misinformation also corresponded. At the uncertain-evidence stage, users of 79.7% (463 out of 581) of posts endorsed the evidence. Within which, 42.5% (197 out of 463) expressed ambiguity about the misinformation, a high percentage comparable with 54.2% (251 out of 463) rejecting the misinformation. In comparison, at the evidence-consensus stage, users of 84.2% (1300 out of 1544) of posts endorsing the evidence rejected the misinformation, and only 10.2% (158 out of 1544) expressed uncertainty about the misinformation. Across the latter two stages, users who rejected or were ambiguous about the evidence hold corresponding attitudes toward the misinformation.

### 3.2. Impacts of Attitude Ambiguity toward the Evidence

**Figure 3:**
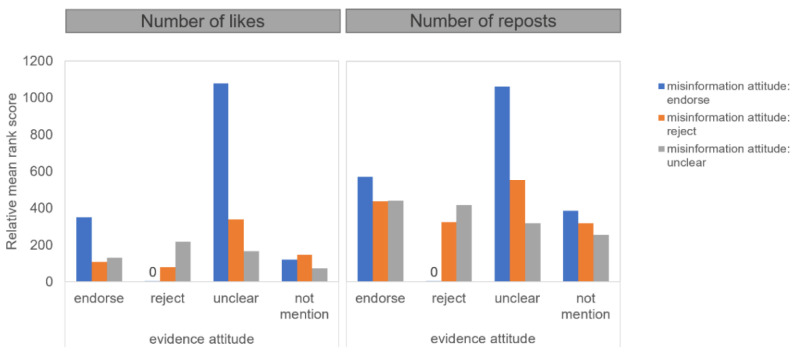
Effects of attitude toward the evidence on numbers of likes and reposts received by the original posts in different attitudes toward the misinformation.

To examine H2, the first test compared numbers of likes and reposts received by original posts in different attitudes toward the evidence and the misinformation. Results from the ART analysis of attitudes toward the evidence in the original posts (four: endorsing vs. rejecting vs. ambiguous vs. not mentioned) * attitudes toward the misinformation in the original posts (three: endorsing vs. rejecting vs. ambiguous) showed that the interaction was significant for both likes (*F*(6, 2488) = 32.3, *p* < 0.001, partial *η^2^* = *0*.072) and reposts (*F*(6, 2488) = 13.3, *p* < 0.001, partial *η^2^* = *0*.031). Figure 3 demonstrates the interaction. Contrast tests further showed that users endorsing the misinformation in posts received more likes and reposts than those rejecting the misinformation when the users also expressed ambiguity toward the evidence in the posts. 

**Figure 4:**
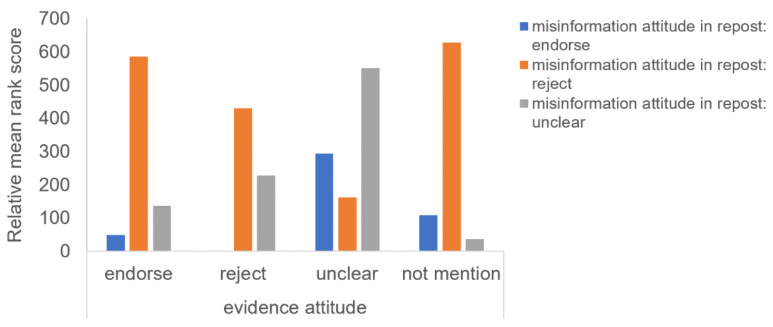
Effects of attitude toward the evidence in the original posts on numbers of reposts in different attitudes toward the misinformation.

The second test compared numbers of reposts in different attitudes toward the misinformation received by original posts in different attitudes toward the evidence. Results from ART analysis on the interaction effects of attitudes toward the evidence in the original posts (between four: endorsing vs. rejecting vs. ambiguous vs. not mentioned) * attitudes toward the misinformation in the reposts (within three: endorsing vs. rejecting vs. ambiguous) showed that the interaction was significant, *F*(6, 1132) = 14.5, *p* < *0*.001. Figure 4 demonstrates the interaction. Contrast tests further showed that original posts where users expressed uncertainty about the evidence received more reposts that repeated endorsement of the misinformation or expressions of uncertainty towards the misinformation, while fewer reposts were received about the rejection of the misinformation, compared with original posts whose authors hold firm attitudes toward the evidence. The two tests above supported H2 that users preferred misinformation to refutations when the original posts expressed ambiguity about the evidence.

### 3.3. Impacts of Evidence Stages

**Figure 5:**
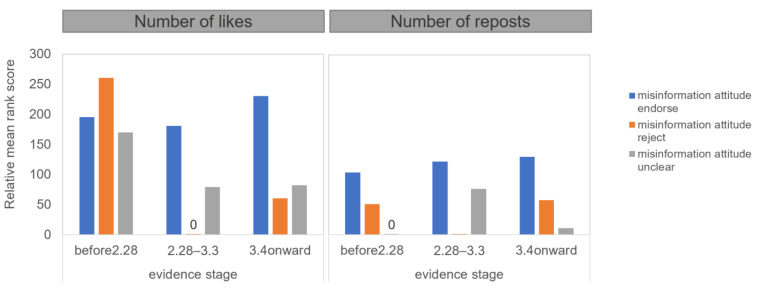
Effects of evidence stage on numbers of likes and reposts received by the original posts.

To examine H3, the first test compared numbers of likes and reposts received by original posts in different attitudes toward the misinformation among different evidence stages. Results from the ART analysis of attitudes toward the misinformation in the original posts (three: endorsing vs. rejecting vs. ambiguous) * evidence stages (three: no evidence, uncertain evidence, evidence consensus) showed that the interaction was significant for both numbers of likes (*F*(4, 2491) = 34.1, *p* < 0.001, partial *η^2^* = 0.052) and reposts (*F*(4, 2491) = 29.9, *p* < 0.001, partial *η^2^*= 0.046). Figure 5 demonstrates the interaction. Contrast tests further showed that posts about the rejection of the misinformation received fewer likes and reposts at the uncertain-evidence stage than those at the other two stages. However, this effect disappeared for posts about the endorsement of the misinformation. 

**Figure 6:**
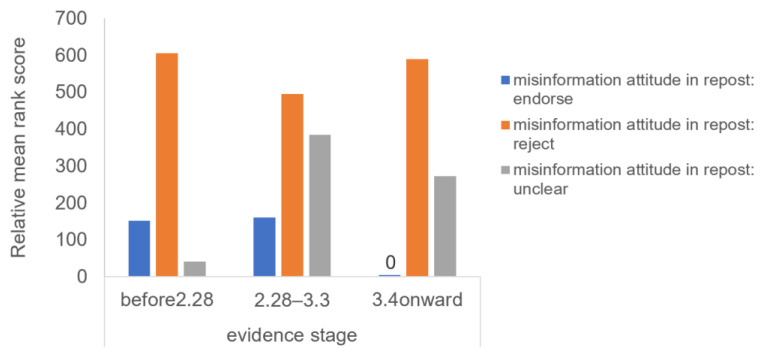
Effects of evidence stage on numbers of reposts with different attitudes toward the misinformation.

The second test compared numbers of reposts with different attitudes toward the misinformation received by original posts published at different stages. Results from ART analysis on the interaction effects of evidence stages (between three: no evidence, uncertain evidence, evidence consensus) * attitudes toward the misinformation in the reposts (within three: endorsing vs. rejecting vs. ambiguous) showed that the interaction was significant, *F*(4, 1134) = 43.4, *p* < 0.001. Figure 6 demonstrates the interaction. Contrast tests further showed that posts published at the uncertain-evidence stage received fewer reposts of rejection of the misinformation and more reposts signaling ambiguity about the misinformation than posts published at the other two stages. The two tests together partly supported H3 by demonstrating that users were averse to refutations, while they did not favor misinformation, at the uncertain-evidence stage.

### 3.4. Interactions between Attitude Ambiguity and Evidence Stages

For the original post analysis, we planned to run analyses of the three-way between-subject interaction effects of attitudes toward the misinformation in the original posts (three: endorsing vs. rejecting vs. ambiguous) * attitudes toward the evidence in the original posts (four: endorsing vs. rejecting vs. ambiguous vs. not mentioned) * evidence stages (three: no evidence, uncertain evidence, evidence consensus) on numbers of likes and reposts. However, in our dataset, there was no instance of posts endorsing the misinformation while signaling evidence ambiguity and published at the uncertain-evidence stage. Similarly, there was no instance of posts rejecting the misinformation while expressing attitude ambiguity and published at the no-evidence stage. Thus, we were unable to test the three-way interactions based on analyses of original posts. 

**Figure 7:**
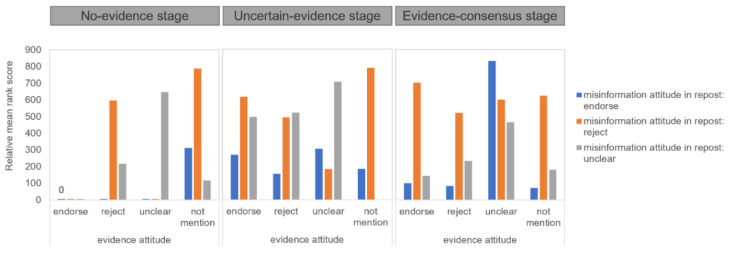
Interaction effects between attitude toward the evidence and evidence stage on numbers of reposts with different attitudes toward the misinformation.

For the repost analysis, we ran the analysis for a three-way mixed effect of attitudes toward the evidence in the original posts (between four: endorsing vs. rejecting vs. ambiguous vs. not mentioned) * evidence stages (between three: no evidence, uncertain evidence, evidence consensus) * attitudes toward the misinformation in the reposts (within three: endorsing vs. rejecting vs. ambiguous) on numbers of reposts. The results showed that the three-way interaction effect was significant, *F*(12, 1116) = 10.5, *p* < 0.001. Figure 7 demonstrates the interaction. Contrast tests further demonstrated that though original posts signaling users’ ambiguous (vs. other) attitudes toward the evidence received more reposts about endorsement of the misinformation at both the uncertain-evidence and the evidence-consensus stage, the effect was much stronger at the later stage. In contrast, the effect that original posts signaling users’ ambiguous (vs. other) attitudes on the evidence received fewer reposts of rejection of the misinformation occurred only at the uncertain-evidence stage.

## 4. Discussion

We set out to examine a new theoretical framework that the uncertainty communicated with preliminary evidence can promote internal motivated reasoning based on a naïve understanding of science that produces misinformation inferred from the evidence. To fulfil the aim, we tested if the communication of preliminary evidence could be a prompt for individuals’ internal processes to generate misinformation and if communicating the uncertainty of the evidence would facilitate misinformation spread based on social media data. We examined evidence uncertainty communication in two forms: users’ ambiguous attitudes toward the evidence and the stage when the evidence was communicated with uncertainty. This study contributes to the literature of science communication and misinformation in several important ways. 

First of all, we gained empirical support for our theoretical framework that the uncertainty circulating around preliminary evidence can promote the generation and transmission of misinformation inferred from the evidence. As the results showed that users’ attitudes toward the evidence and the misinformation corresponded, this suggested that users indeed perceived an inherent link between the evidence and the misinformation based on their naïve understanding of science. Importantly, users’ ambiguous attitudes toward the evidence and the uncertain-evidence stage resulted in more likes and retweets of the misinformation and/or fewer likes and retweets of the refutations. This further indicates that the uncertainty signaled in the posts strengthens individuals’ beliefs in such an inherent link. This is likely because the uncertainty prompts individuals to seize and freeze an available short-cut to reduce the uncertainty. However, the current data were unable to demonstrate such a mechanism, and future experimental studies are required. Nevertheless, this study indicates that health policymakers should at least regulate the hasty communication of emerging evidence with inherent uncertainty during a novel health crisis for the purpose of misinformation control.

Second, our study highlights and extends Davis and Loftus’ framework [13] on internal sources of misinformation that assumes an exposure to accurate information by focusing on an exposure to preliminary information. Future studies should continue this line of research by examining other possible internal sources of misinformation. For example, Lu found concurrence between the announcement of the Wuhan lockdown in early 2020 and the rise of fake news about government quarantine policies in China [2]. It seems clear that there is another undiscovered internal cognitive process that causes misinformation after individuals’ exposure to the factual information of the city lockdown.

Third, this study revisits Rosnow’s proposal [19] on examining information contexts that feed and fuel the misinformation. Our data supported that, besides misinformation and individual characteristics, contextual factors such as evidence uncertainty can promote misinformation. Together with the proposal, this study suggests future research on misinformation prevention strategies. This suggestion goes beyond corrections that are extensively studied in the current literature by advocating to build a misinformation-unfriendly context through ways such as strategic social media use and evidence framing, some of which will be discussed below.

Extending previous research, our investigation revealed that Weibo served as a platform that promoted misinformation and inhibited the propagation of refutations when the emerging evidence was communicated with uncertainty. Particularly, we found that authors endorsing the misinformation while expressing ambiguous attitudes toward the evidence in their posts received more likes and retweets than those rejecting or endorsing the evidence. In addition, users tended not to share the refutation posts when the evidence was first released and had not gained any consensus. These findings suggest that Weibo-like social media platforms may not be suitable for communicating uncertainty toward early evidence during novel crises. They warrant policy attention as studies have suggested a high prevalence in highlighting scientific uncertainty associated with COVID-19 preprints on digital media outlets [10]. In addition, the interaction analysis between attitude ambiguity and the evidence stage further revealed that posts did not mention the evidence received sizeable refutation reposts across different evidence stages. This implies that when preliminary evidence is released, an appropriate communication practice is not to express uncertainty about the evidence but focus only on debunking the inferred misinformation. 

Interestingly, and in contrast, we found that Weibo might be good for communicating scientific consensus. The interaction analysis between attitude ambiguity and the evidence stage revealed that users tended to debunk the misinformation in their reposts when the original posts supported the evidence consensus. In comparison, if the original posts signaled uncertainty about the evidence consensus, users tended to spread the misinformation instead. These findings are consistent with the well-documented knowledge that the communication of consensus uncertainty will lead to the endorsement of one’s heuristic beliefs in the inferred (mis)information [38]. Nevertheless, contrary to previous experimental findings that consensus uncertainty could also lead to people disregarding authorities’ recommendations, we observed sizable refutations following such communication of consensus uncertainty. A closer examination of those refutations revealed that they were trying to restore the consensus and fight the misinformation. This suggests that building public consensus on scientific evidence may help tackle relevant misinformation on social media [48].

In general, we found that expressing attitude ambiguity toward the evidence at different evidence stages was associated with different patterns in reposts of misinformation and refutation messages. Our analyses revealed that attitude ambiguity toward the evidence suppressed the dissemination of refutations only at the uncertain-evidence stage, but not at the evidence-consensus stage. In contrast, such ambiguity promoted misinformation to a greater extent at the later stage than at the former stage. A possible explanation of these findings may be that attitude ambiguity at different evidence stages may signal different levels and types of evidence uncertainty. For example, attitude ambiguity toward a piece of uncertain evidence may signal a strong deficient uncertainty about a known gap, and thus the evidence should be less convincible for rebuttals [31]. In comparison, attitude ambiguity toward the evidence consensus should signal consensus uncertainty and induce the adoption of misinformation [32]. Future research should explore the social–cognition mechanisms underlying these findings. 

In this study, we included data before any evidence emerged in the analysis. At first glance, the data seem not so relevant to our research question. However, we think that they are of both theoretical and practical significance. Theoretically, these data provided a baseline for comparison to demonstrate the effect of communicating emerging evidence related to misinformation. We showed that original posts about the rejection of the misinformation received a decreased number of likes after the evidence emerged. Such a decrease was greater at the uncertain-evidence stage than at the evidence-consensus stage. In addition, those original posts received a decrease in rejection reposts on the misinformation only at the uncertain-evidence stage, but not at the evidence-consensus stage. These findings suggest that the effect was associated with evidence uncertainty rather than evidence consensus. 

Practically, analyses on data from the no-evidence stage showed that posts that induced uncertainty about a piece of “fake” evidence would suppress the dissemination of misinformation rebuttals and promote ambiguous beliefs on the misinformation. This finding provides empirical support on how misinformation can be spread with groundless evidence [49]. Our analyses also revealed that the refutation of such groundless evidence can be a good way to tackle misinformation spread as it can drive propagations of debunking messages. 

This study has several limitations. First, we were not able to measure users’ cognitive processes using social media data. Second, we examined our hypotheses with only a circumstance of misinformation, which may be a constraint in result generalization. Third, limited by the method of ART analysis, we were not able to include any covariate to control for potential impacts of other relevant factors, such as the emotional tone of the posts and account attributes. Finally, Weibo is a Twitter-like social media platform where users’ relationships are asymmetrical and information is open and prosperous. It is quite different from Facebook and WeChat, where social interactions occur mainly among closed relationships and information is often private and exclusive. Therefore, comparisons between Weibo and other platforms are needed. 

## 5. Conclusions

In summary, our study provides the first empirical support that misinformation can be induced and spread on social media because of the communication of uncertainty about the emerging scientific evidence during a novel health crisis. Supporting H1, our results showed that attitudes toward the misinformation and the evidence were associated, suggesting that the communication of preliminary evidence could induce misinformation. Our findings also supported H2 that users preferred misinformation to refutations when the original posts expressed ambiguity about the evidence. The findings partly supported H3 by demonstrating that users were averse to refutations at the uncertain-evidence stage. Finally, our results answered the RQ by revealing that users’ attitudes toward the evidence and the stage when the evidence was communicated interactively affected misinformation spread. Our study extends the frameworks of internal sources of misinformation by demonstrating that a naïve understanding of scientific evidence can be a source of misinformation. Additionally, this study revisits the contextual perspective of misinformation and supports that contextual factors are important drivers of misinformation generation and spread. Finally, this study suggests to build a misinformation-unfriendly environment that prevents the generation and spread of misinformation. Particularly, our study suggests that health policymakers should regulate the hasty communication of emerging evidence during a novel health crisis. In addition, authorities should better use Weibo-like social media to communicate scientific consensus rather than uncertainty about the emerging evidence.

## Figures and Tables

**Table 1:** The crosstab frequency table of users’ attitudes toward the misinformation and its evidence in original posts.

			Pets Can Be Infected			
			Not Mentioned	Endorse	Reject	Unclear
Overall	pets transmit COVID-19 to humans	not mentioned	0	80	18	9
		endorse	43	46	2	2
		reject	287	1552	91	3
		unclear	55	356	27	36
Before 28 February	pets transmit COVID-19 to humans	not mentioned	0	7	4	1
		endorse	22	18	2	1
		reject	107	1	52	0
		unclear	21	1	1	7
28 February to 3 March	pets transmit COVID-19 to humans	not mentioned	0	8	5	1
		endorse	5	7	0	0
		reject	69	251	14	1
		unclear	4	197	7	12
4 March onward	pets transmit COVID-19 to humans	not mentioned	0	65	9	7
		endorse	16	21	0	1
		reject	111	1300	25	2
		unclear	30	158	19	17

Note. N = 2607.

## Data Availability

The data presented in this study are available on request from the corresponding author. The data are not publicly available due to strict data policy by the funders.
